# The Effect of Impact Load on the Atomistic Scale Fracture Behavior of Nanocrystalline bcc Iron

**DOI:** 10.3390/nano14040370

**Published:** 2024-02-16

**Authors:** Zhifu Zhao, Zhen Wang, Yehui Bie, Xiaoming Liu, Yueguang Wei

**Affiliations:** 1 The State Key Laboratory of Nonlinear Mechanics (LNM), Institute of Mechanics, Chinese Academy of Sciences, Beijing 100190, China; 2 School of Engineering Science, University of Chinese Academy of Sciences, Beijing 100049, China; 3 Department of Mechanics and Engineering Science, College of Engineering, Peking University, Beijing 100871, China

**Keywords:** molecular dynamics simulation, nanocrystalline iron, impact load, fracture resistance, fracture ductility

## Abstract

Nanocrystalline metals have many applications in nanodevices, especially nanoscale electronics in aerospace. Their ability to resist fracture under impact produced by environmental stress is the main concern of nanodevice design. By carrying out molecular dynamics simulations under different fast loading rates, this work examines the effect of impact load on the fracture behavior of nanocrystalline bcc iron at an atomistic scale. The results show that a crack propagates with intergranular decohesion in nanocrystalline iron. With the increase in impact load, intergranular decohesion weakens, and plastic behaviors are generated by grain boundary activities. Also, the mechanism dominating plastic deformation changes from the atomic slip at the crack tip to obvious grain boundary activities. The grain boundary activities produced by the increase in impact load lead to an increase in the threshold energy for crack cleavage and enhance nanocrystalline bcc iron resistance to fracture. Nanocrystalline bcc iron can keep a high fracture ductility under a large impact load.

## 1. Introduction

Nanocrystalline metals have many applications in nanodevices due to their good physicochemical properties [1,2,3]. Nanodevices, especially nanoscale electronics in aerospace, usually require high impact resistance. The ability of nanocrystalline metals to resist fracture under impact produced by environmental stress is the main concern of nanodevice design. Revealing the fracture behaviors and mechanisms of nanocrystalline materials is an important prerequisite for improving fracture resistance and evaluating service safety. Under an impact produced by environmental stress, nanodevices are subjected to stress with a large growth rate. The stress growth rate is determined by the magnitude of the impact load. The dependence of fracture behavior on stress growth rate is,, therefore, of great research interest. Experiments on polycrystalline metals have shown that the stress growth rate has significant effects on yield strength [4,5], tensile strength [5,6], fracture toughness [7,8,9], crack tunneling [10], and crack growth speed [11]. Different from polycrystalline metals, nanocrystalline metals have a large grain boundary proportion. The activities of grain boundaries and cracks and the interactions between them can seriously affect the fracture behavior of nanocrystalline metals. As a result, the fracture behavior of nanocrystalline metals under an impact produced by environmental stress may be significantly different from that of polycrystalline metals.

Atomic-scale techniques, such as in situ experiments and molecular dynamics simulations, can reveal the details of activities of grain boundaries and cracks and the interactions between them during nanocrystalline metal fractures. In situ experiments usually require high-level operations for researchers, and the observed samples generally have defects and impurities [12]. Compared with in situ experiments, molecular dynamics simulations are more accessible and efficient. By carrying out molecular dynamic simulations on nanocrystalline metals without pre-existing cracks, researchers observed grain boundary migration as well as diffusion and dislocation nucleation and motion during nanocrystalline metal fractures [13,14,15,16,17]. For nanocrystalline metals with larger grain sizes, dislocation nucleation and motion are more obvious than grain boundary migration and diffusion [17,18,19]. The deformation of nanocrystalline metals is dominated by dislocation activities. With a decrease in grain size, a change in the dominant deformation mechanism from dislocation nucleation and motion-to-grain boundary migration and diffusion appears [17,18,20,21,22,23]. This change also results in the transition of the relationship between tensile strength and grain size from Hall–Petch to inverse Hall–Petch. Simulations showed that grain boundary mobility can be reduced by adding alloy atoms to improve the deformation behavior of nanocrystalline metals, such as doping Zr atoms at the grain boundaries in nanocrystalline Cu [24]. The activities of dislocation and grain boundaries can cause the breakage of atomic bonds and nanovoid nucleation at grain boundaries, particularly at the triple junctions [25,26,27]. The coalescence of nanovoids generates nanocracks. Nanocracks propagate along grain boundaries, which causes intergranular fractures [27,28,29]. Nanocrack initiation and propagation were observed to preferably take place at the high-angle grain boundaries perpendicular to the stretching direction [29]. Different from the fracture behavior of nanocrystalline metals without pre-existing cracks, dislocation and twinning are generated around crack tips besides grain boundary activities during the fracture of nanocrystalline metals with pre-existing cracks [30,31,32,33,34]. With crack propagation in nanocrystalline metals, nanovoids are usually generated at grain boundaries ahead of the crack [33]. A further study by the authors showed that these nanovoids are generated by intergranular decohesion that results from the fast Intergranular crack propagation on some cross-sections, and the fracture behavior of cracked nanocrystalline metals is dominated by crack propagation instead of grain boundary activities [34]. However, the high grain boundary proportion can indeed significantly improve the fracture toughness of nanocrystalline metals [32,33,34]. This improvement was found to be attributed to the weakening of intergranular decohesion [34].

As mentioned above, researchers obtained some valuable results regarding the fracture behavior of nanocrystalline metals by focusing on the activities of grain boundaries and cracks and the interaction between them. However, the competition between crack propagation and grain boundary activities under an impact environment remains unclear. Due to the requirements to achieve high saturation magnetic flux density with low losses and respond quickly to changes in external magnetic fields of the nanoscale electronics in aerospace, nanocrystalline bcc iron and its alloys with excellent soft magnetic properties have been developed and widely used [35,36,37]. The fracture failure of nanocrystalline iron has a significant effect on the reliability of nanoscale electronics. Hence, the authors of this work develop a large nanocrystalline bcc iron model with a pre-existing crack and investigate its fracture behavior under stretch loads with different fast growth rates by carrying out molecular dynamics simulations. The competition between crack propagation and grain boundary activities under impacts produced by environmental stress is fully discussed. It is believed that a better understanding of the nature of the large loading rate effect on nanocrystalline iron can be useful in preventing catastrophic failures of nanoscale electronics in aerospace when encourterning impacts produced by environmental stress.

## 2. Model and Methods

**Figure 1:**
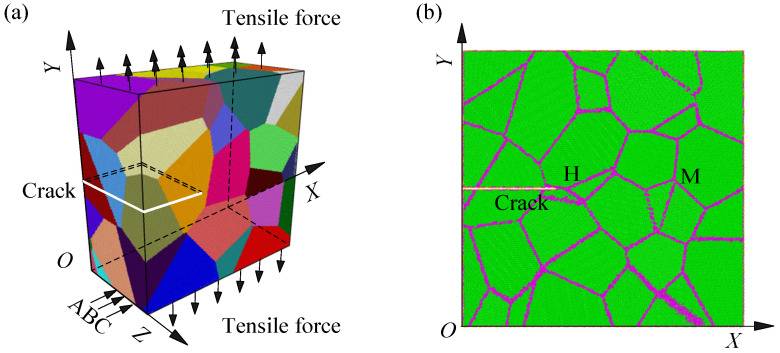

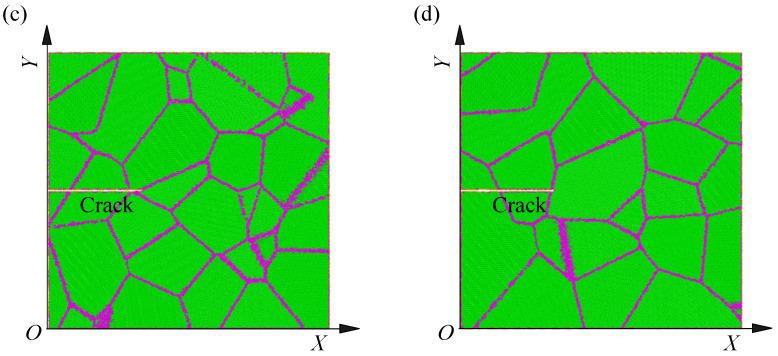
Nanocrystalline bcc iron model. (**a**) shows a 3D view, where colors are used to distinguish different grains. (**b**–**d**) show the distributions of grain boundaries and atoms on different cross-sections (A, B, and C) normal to *Z* direction, where the *Z* values of atoms on cross-section A are between 14.78 and 15.22 nm. Those on cross-section B are between 16.78 and 17.22 nm, and those on cross-section C are between 23.78 and 24.22 nm. In (**b**–**d**), green atoms have bcc crystal structure and are located inside the grains; pink ones are located at grain boundaries, and yellow ones are located at the surfaces of crack and model.

In this work, a nanocrystalline bcc iron model with average grain diameter of 15 nm was developed by Voronoi method, where the crystal orientation of each grain is random, as shown in Figure 1a. The Voronoi method was shown to produce nanocrystals with grain boundary structures similar to what is expected in polycrystalline material and log-normal grain size distributions similar to what is found in experiments [14,31] and is, therefore, widely used to construct the atomistic models [14,15,16,17,18,19,20,23,24,25,26,27,28,30,31,32,34]. The developed model has *L_X_* × *L_Y_* × *L_Z_* dimensional size of 60 nm × 60 nm × 30 nm and 9,197,099 iron atoms. It is very isotropic and has a good inverse Hall–Petch relationship with nanocrystalline irons with average grain diameters of 5 nm and 10 nm [34]. After developing the nanocrystalline model, the processes of minimization, equilibration, and stretching simulation are performed in a large-scale atomic/molecular massively parallel simulator [38]. These processes are based on the precise description of the interactions between iron atoms. In this work, the embedded-atom potential developed by Mendelev et al. [39] is applied. During minimization, the conjugate gradient algorithm is used. The energy and force tolerances are 10^−15^, and the maximum number of force and energy evaluations is 10^4^. Due to the large dimensional size, minimization process is performed many times until the model state parameters remain unchanged. After minimization, an edge crack with front parallel to *Z* direction is created at the left of the model by directly deleting atoms, and its length in *X* direction is 20 nm. In *Y* direction, the crack is the same distance from the top and bottom of the model, as shown in Figure 1. Then, atomic initial velocities are set by a random number generator with the specified seed at the temperature of 1 K to avoid thermal effect. Based on atomic locations at the end of minimization and atomic initial velocities, the cracked model is fully equilibrated under microcanonical ensemble, where atomic velocities are explicitly rescaled to remain at a temperature of 1 K. After equilibration, the cracked model is stretched along *Y* direction by adding force on several layer atoms on the top and bottom under microcanonical ensemble, as shown in Figure 1a. In the directions of *X* and *Y*, free boundaries are applied to stretch the cracked model freely. In the directions of *Z*, periodic boundaries are applied to avoid the effect of extra surface on crack propagation. Additionally, the application of periodic boundaries puts the cracked model in a plane strain state. During the processes of equilibration and stretching simulation, velocity Verlet algorithm [40] is used to solve Newton’s equations, and timestep is 10^−3^ ps. To identify the effect of loading rate on crack propagation in nanocrystalline bcc iron, the stretch process is performed five times, and the growth rates of stretch stress are 6.65 × 10^−2^ Gpa/ps, 1.33 × 10^−1^ Gpa/ps, 2.00 × 10^−1^ Gpa/ps, 2.66 × 10^−1^ Gpa/ps, and 3.33 × 10^−1^ Gpa/ps, respectively. As shown in Figure 1b–d, the distribution of grain boundaries and crystal orientation of grains are very different on different cross-sections normal to *Z* direction. Hence, the crack propagation behaviors on different cross-sections are very different. Discussion on the loading rate effect needs to consider crack propagation behaviors on different cross-sections.

## 3. Results and Discussions

### 3.1. Engineering Stress–Strain Behaviors

**Figure 2:**
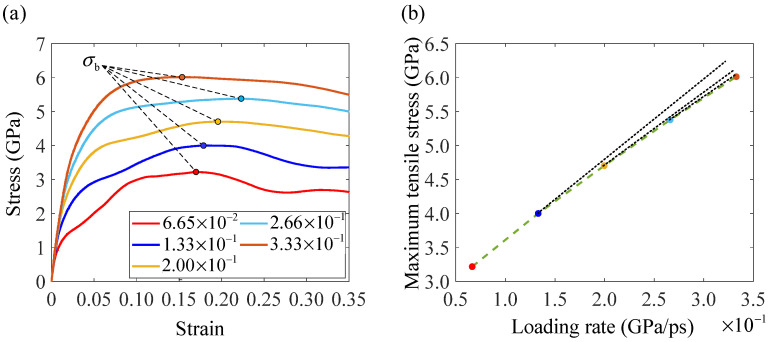
Engineering stress–strain behaviors under different loading rates, where (**a**) is the stress–strain curves and (**b**) is the maximum tensile stresses. The dashed lines in (**b**) are drawn linearly based on two adjacent points.

Engineering stress–strain curve is a direct way to learn about the fracture behavior of nanocrystalline bcc iron. Under different loading rates, the engineering stress–strain behaviors of the cracked nanocrystalline bcc iron model varies greatly, as shown in Figure 2a. The ultimate tensile stress $\sigma_{b}$ increases with the increase in loading rate in a weak nonlinear way. In other words, the second derivative of ultimate tensile stress with respect to loading rate is negative, which is consistent with the experimental results on steels without pre-existing cracks [41]. Plastic deformation becomes more significant with an increase in the loading rate. However, the shapes of stress–strain curves under loading rates lower than 2.00 × 10^−1^ GPa/ps are very different from those under loading rates higher than 2.00 × 10^−1^ GPa/ps. When the loading rate is lower than 2.00 × 10^−1^ GPa/ps, secondary hardening can be observed clearly. However, when the loading rate is higher than 2.00 × 10^−1^ GPa/ps, secondary hardening cannot be observed. Therefore, the mechanisms of plastic deformation under loading rates lower than 2.00 × 10^−1^ GPa/ps may be different from those under loading rates higher than 2.00 × 10^−1^ GPa/ps. The different plastic deformation mechanisms can lead to the obvious difference in fracture behavior of the cracked nanocrystalline model.

### 3.2. Crack Propagation Behaviors

**Figure 3:**
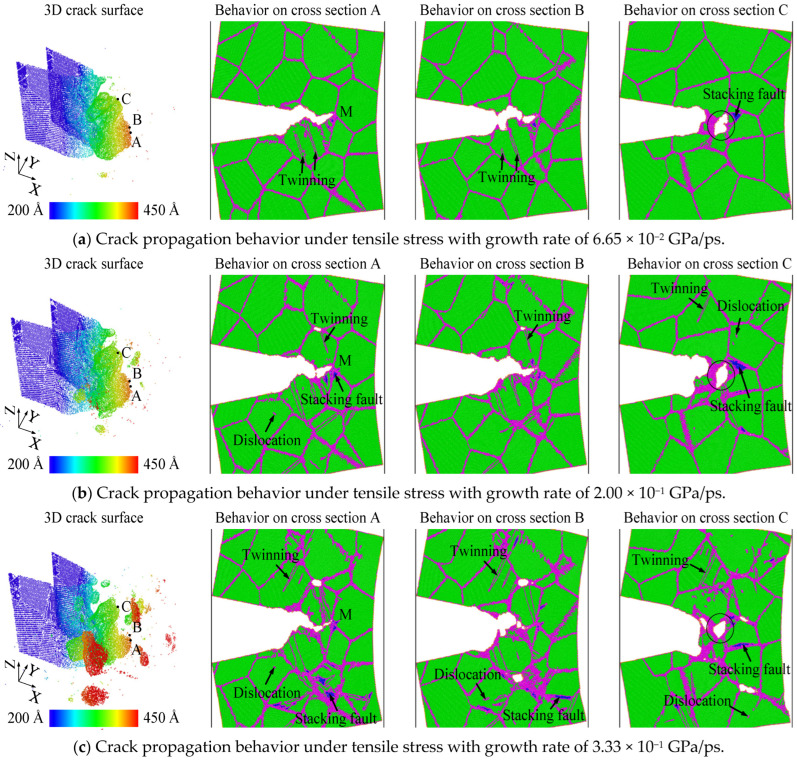
Crack propagation behaviors in nanocrystalline bcc iron under tensile stress with different growth rates, where cross-sections A, B, and C are atomic planes that contain points A, B, and C in 3D view, respectively. In the figure of 3D crack surface, the color gradient is on the basis of *X* coordinate value of iron atom. In the figures that depict behaviors on cross-sections A, B, and C, green and blue atoms have bcc and fcc crystal structures, respectively; pink ones are located at grain boundaries and dislocation cores, and yellow atoms are located at the surfaces of crack and voids.

Figure 3 shows the crack propagation behaviors under different loading rates by visualization software OVITO (https://www.ovito.org/) developed by Stukowski [42], where the behaviors on different cross-sections are presented. During crack propagation, plastic behaviors, including stacking faults, twinning bands, and dislocations, can be generated. The common neighbor analysis [43], nearest neighbor number [44], centro-symmetry parameter [45], and atomic potential energy are calculated to identify the atoms correlated to plastic behaviors and differentiate them from surface atoms and bcc iron atoms. Details can be found in a previous study [34]. To examine the loading rate effect, the state of crack propagation when the crack tip first reaches the grain boundary junction of ‘M’ on section A is used as the criterion. Thus, the crack propagation behaviors in Figure 3a–c are at 40.4 ps, 23.8 ps, and 19.5 ps, respectively. As shown in Figure 3, brittle cleavage without obvious plastic deformation and ductile propagation with obvious plastic deformation can occur on different cross-sections. Fast brittle cleavage on some cross-sections can accelerate ductile propagation on their adjacent cross-sections through intergranular decohesion. For example, the void marked by a solid circle on cross-section C forms at grain boundaries and connects with the crack surface on cross-section A in Figure 3. The three-dimensional crack surface clearly describes the relationship between the marked void in section C and the crack tip in section A. From cross-section A to cross-section C, the void surface coincides with the grain boundaries, and the void size decreases gradually. Hence, the marked void is generated by the extension of fast cleavage on cross-section A along grain boundaries to cross-section C. This extension is a typical intergranular decohesion process. The marked void growth consumes the accumulated plastic deformation generated by crack transgranular propagation, and its convergence with the main crack can accelerate crack ductile propagation on cross-section C. With an increase in loading rate, the size of the void on cross-section C decreases, as well as the portion of the cross-section where fast cleavage occurs. As a consequence, the effect of intergranular decohesion weakens with the increasing loading rate. Additionally, one can find that more plastic behaviors are generated by grain boundary activities when the loading rate is increasing. The accumulation of this plastic deformation causes the formation of microvoids at the position far away from the crack tip. Because these microvoids are far away from the main crack, they cannot converge with the main crack. Plastic deformation and microvoid formation consume a lot of energy, so they seriously hinder the main crack propagation. The significant grain boundary activities are the main reasons for the weakening intergranular decohesion and high fracture toughness for a high loading rate. The fracture mechanism of nanocrystalline bcc iron is very different from that of coarse-grained iron under a high loading rate. Experimental results show that the fracture behavior of coarse-grained iron is determined by the twinning onset and dislocation motion at the crack tip under high loading rates [46]. There is no evidence that grain boundaries far from the crack tip provide sites for plastic deformation during the fracture process of coarse-grained iron specimens with a sharp crack. The high fracture toughness induced by obvious plastic deformation at grain boundaries in nanocrystalline bcc iron under high loading rates is instead similar to the fracture characteristics of metals or alloys with positive $k_{\varepsilon_{f}}$ under quasi-static loading, where $k_{\varepsilon_{f}}$ is an experimental constant indicating the relationship between fracture toughness and average grain size [47]. For metals or alloys with positive $k_{\varepsilon_{f}}$, grain refinement allows grain boundaries to offer sites for plastic deformation, which increases the energy expensed during ductile fracture and promotes fracture toughness. The differences in crack propagation behavior and grain boundary activity under different loading rates lead to differences in stress–strain behavior. When the loading rate is lower than 2.00 × 10^−1^ GPa/ps, crack propagation dominates the stress–strain behavior. The obvious ductile transgranular propagation can result in secondary hardening. When the loading rate is higher than 2.00 × 10^−1^ GPa/ps, grain boundary activities hinder crack propagation and dominate the stress–strain behavior. The fracture behavior of nanocrystalline bcc iron keeps high toughness due to the obvious grain boundary activities. Hence, secondary hardening cannot be observed.

### 3.3. Ductile–Brittle Characteristics

**Figure 4:**
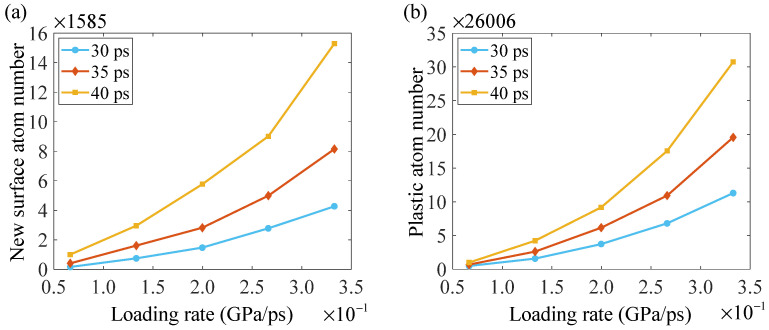
Variations of (**a**) new surface atom number and (**b**) plastic atom number during crack propagation under different loading rates.

To further describe the effect of loading rate on the ductile–brittle characteristics of crack propagation, a quantitative investigation is carried out. The atoms with fcc, hcp, and ico crystal structures, and the atoms that are located at the dislocation cores and the boundaries of stacking faults and twinning bands, are defined as plastic atoms. The new surface atom number and the plastic atom number can be used to quantitatively describe crack propagation behavior. Figure 4 gives the new surface atom number and the plastic atom number at three random moments for each simulation. As shown in Figure 4, the difference between the new surface atom number at 40 ps and 35 ps is much larger than that between the new surface atom number at 35 ps and 30 ps for each simulation. Therefore, the new surface atom number increases in a strong nonlinear way for each simulation; thus, its second derivative with respect to time is positive. The variation of the plastic atom number is similar to that of the new surface atom number. With an increasing loading rate, the new surface atom number and the plastic atom number also increase in a strong nonlinear way. However, their growth speeds have a significant difference. For example, the new surface atom number under the loading rate of 3.33 × 10^−1^ GPa/ps is 15.3 times that under the loading rate of 6.65 × 10^−2^ GPa/ps, while the plastic atom number under the loading rate of 3.33 × 10^−1^ GPa/ps is 30.74 times that under the loading rate of 6.65 × 10^−2^ GPa/ps. With an increase in loading rate, the plastic atom number grows faster than the new surface atom number.

According to fracture mechanics, the energy required to form a unit area of crack unilateral surface is usually used to measure the resistance to crack growth. For ductile fracture, the required energy contains the energy $G_{s}$ consumed to form a crack surface and the energy $G_{p}$ consumed to generate plastic deformation; thus

(1) $$G_{d}=G_{s}+G_{p}$$

During new crack surface formation and plastic deformation generation, atomic kinetic and potential energies will increase. Compared with the increment of atomic potential energy, the increment of atomic kinetic energy is small and can be ignored. Hence, the increment of atomic potential energy is usually used to measure the resistance to crack growth [31]. Supplementary Figure S1 also shows that the change of atomic kinetic energy is related to the crack surface movement, but the change of atomic potential energy is related to the new crack surface formation and plastic deformation generation. Therefore, the calculations for $G_{s}$ and $G_{p}$ in this work only consider the increment of atomic potential energy. For the nanocrystalline model with a pre-existing crack, the value of *G*_p_ can be very large in the initial stage of crack cleavage because plastic behaviors can be generated before cleavage on most cross-sections. To avoid this situation, the term of *G*_p_ can be written as

(2) $$G_{p}=\frac{\sum_{i=1}^{N_{p}}\Delta E_{i}^{p}}{A_{0}+N_{s}/\left(2\rho_{s}\right)}$$

where $N_{p}$ is the plastic atom number, $\Delta E_{i}^{p}$ is the extra potential energy that the *i*th atom gains when it participates in plastic deformation, $A_{0}$ is the initial area of crack unilateral surface, $N_{s}$ is the new surface atom number, and $\rho_{s}$ is the surface atom number on a unit area. According to the pre-existing crack, the value of $\rho_{s}$ is calculated as 10.173/nm^2^. Similarly, the term of *G*_s_ can be computed as

(3) $$G_{s}=\frac{\Gamma_{0}+\sum_{j=1}^{N_{s}}\Delta E_{j}^{s}}{A_{0}+N_{s}/\left(2\rho_{s}\right)}$$

where $\Gamma_{0}$ is the total potential energy of atoms on the initial surface of a pre-existing crack, and $\Delta E_{j}^{s}$ is the extra potential energy that the *j*th atom gains when it becomes a surface atom.

**Figure 5:**
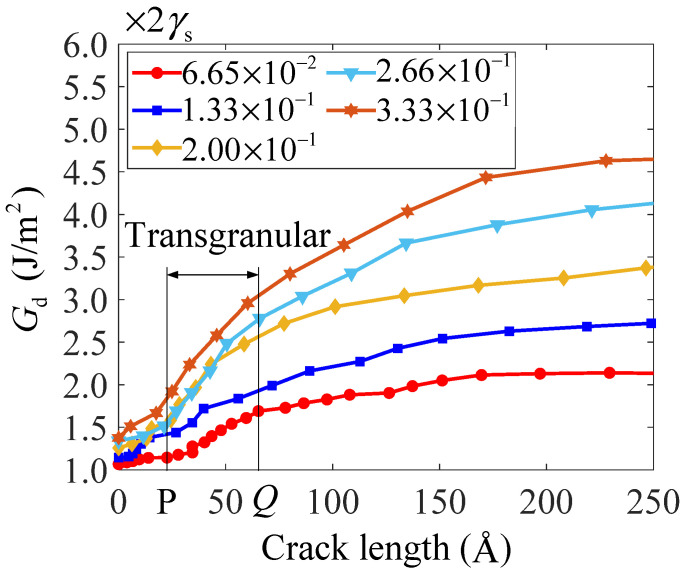
Variations of the energy required to form a unit area of crack unilateral surface with crack growth length under different loading rates.

As shown in Figure 5, the energy required to form a unit area of crack unilateral surface increases with crack growth, which conforms to the ductile fracture characteristic of nanocrystalline bcc iron. When the crack starts to cleave, the threshold energy for crack cleavage increases with the loading rate increasing. However, no plastic behavior is generated at the same time in each simulation because the crack first cleaves in an intergranular way on some cross-sections, for example, cross-section A in Figure 1b. Before crack cleavage, the increasing number of atoms at grain boundaries is observed, and the decreasing number of atoms with bcc crystal structure is observed. With the increasing loading rate, the number of atoms at grain boundaries increases significantly before crack cleavage. Therefore, the increase in loading rate can promote the activity of grain boundaries in nanocrystalline bcc iron, which results in an increase in the threshold energy for crack cleavage and enhances the resistance to the fracture of nanocrystalline bcc iron. When the crack arrives at the grain boundary junction of ‘H’ on cross-section A, the intergranular crack propagation will be transformed into transgranular crack propagation at a small, limited distance. At the same time, cracks also propagate in a transgranular way on other cross-sections. Hence, the required energy *G*_d_ increases significantly with crack growth at this stage, as shown by the portion designated by PQ in Figure 5. When the loading rate is lower than 2.00 × 10^−1^ GPa/ps, crack propagation behavior is more obvious than grain boundary activities. The increase in loading rate can lead to an obvious increase in the growth rate of the required energy *G*_d_. With the loading rate further increasing, grain boundary activities become more and more obvious. As a result, the growth rate of the required energy *G*_d_ cannot increase significantly, with the loading rate further increasing when the loading rate is more than 2.00 × 10^−1^ GPa/ps. Out of the stage designated by PQ, intergranular crack propagation occurs again on some cross-sections, and it will accelerate crack ductile growth on other cross-sections by intergranular decohesion. With the loading rate increasing, the intergranular decohesion weakens. The difference in the required energy *G*_d_ under different loading rates becomes obvious. When crack growth length is 250 Å, the required energy *G*_d_ under the loading rate of 3.33 × 10^−1^ GPa/ps is almost 2.62 times that under the loading rate of 6.65 × 10^−2^ GPa/ps and almost 4.7 times the Griffith energy release rate. In this work, the Griffith energy release rate is measured as 3.58 J/m^2^. The fracture with high required energy *G*_d_ has a high ductility. The increase in loading rate can significantly increase the fracture ductility of nanocrystalline bcc iron.

## 4. Conclusions

This work investigates the atomistic scale fracture behaviors of cracked nanocrystalline bcc iron under different loading rates by carrying out molecular dynamics simulations. The main conclusions are as follows:

(1) The engineering stress–strain behaviors of cracked nanocrystalline iron are very disparate under different loading rates. On the one hand, the ultimate tensile stress increases with an increase in loading rate in a weak, nonlinear way. On the other hand, plastic deformation will cause secondary hardening of the stress–strain behavior at a low loading rate but not at a high loading rate.
(2) Ductile–brittle characteristics of crack propagation on different cross-sections are very unique, and fast brittle cleavage on some cross-sections can accelerate ductile propagation on their adjacent cross-sections through intergranular decohesion. With the loading rate increasing, intergranular decohesion weakens, and more plastic behaviors are generated by grain boundary activities. The obvious grain boundary activities result in the formation of microvoids at the position far away from the crack tip and hinder crack propagation. The differences in crack propagation behavior and grain boundary activity under different loading rates determine whether secondary hardening occurs or not.
(3) The promoted grain boundary activities by the increase in loading rate cause an increase in the threshold energy for crack cleavage and enhance the resistance to the fracture of nanocrystalline bcc iron. After crack cleavage, both the new surface atom number and the plastic atom number grow with an increase in loading rate in a strong, nonlinear way, but the plastic atom number grows faster than the new surface atom number. The increase in loading rate can significantly increase the fracture ductility of nanocrystalline bcc iron.

By applying stretch loads with different fast growth rates, this work provides a reference to reveal the fracture behaviors of nanocrystalline metals under different impact environments. The results have important implications for the safety design of related nanodevices.

## Data Availability

The data that support the findings of this study are available from the corresponding author upon reasonable request.

## Supplementary Materials

The following supporting information can be downloaded at: https://www.mdpi.com/article/10.3390/nano14040370/s1, Figure S1: The distributions of atomic kinetic and potential energies.
