# Supplementary material for: The Effect of Impact Load on the Atomistic Scale Fracture Behavior of Nanocrystalline bcc Iron
Source: Nanomaterials (Basel). 2024 Feb 16;14(4):370. doi: 10.3390/nano14040370 (PMC10891635; doi:10.3390/nano14040370)
Supplement: Supplementary file 1 [file nanomaterials-14-00370-s001.zip › nanomaterials-2822649-supplementary.pdf]

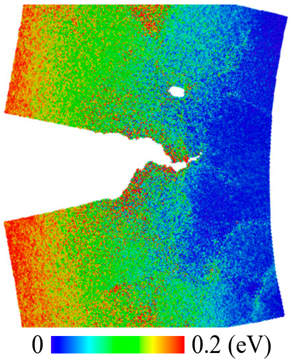

(a) Atomic kinetic energy distribution on cross section A at 19.5 ps under tensile stress with growth rate of 3.33×10^{-1} GPa/ps.

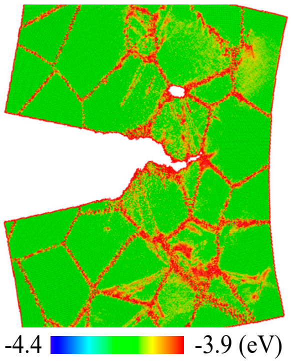

(b) Atomic potential energy distribution on cross section A at 19.5 ps under tensile stress with growth rate of 3.33×10^{-1} GPa/ps.
